# Accurately Models the Relationship Between Physical Response and Structure Using Kolmogorov–Arnold Network

**DOI:** 10.1002/advs.202413805

**Published:** 2025-02-07

**Authors:** Yang Wang, Changliang Zhu, Shuzhe Zhang, Changsheng Xiang, Zhibin Gao, Guimei Zhu, Jun Sun, Xiangdong Ding, Baowen Li, Xiangying Shen

**Affiliations:** ^1^ State Key Laboratory for Mechanical Behavior of Materials School of Materials Science and Engineering Xi'an Jiaotong University Xi'an 710049 P. R. China; ^2^ Department of Physics Southern University of Science and Technology Shenzhen 518055 P. R. China; ^3^ Department of Materials Science and Engineering Southern University of Science and Technology Shenzhen 518055 P. R. China; ^4^ School of Microelectronics Southern University of Science and Technology Shenzhen 518055 P. R. China; ^5^ Shenzhen International Quantum Academy Shenzhen 518017 P. R. China

**Keywords:** Kolmogorov‐Arnold Network, Poisson's ratio, Mechanical property, Deep learning

## Abstract

Artificial intelligence (AI) in science is a key area of modern research. However, many current machine learning methods lack interpretability, making it difficult to grasp the physical mechanisms behind various phenomena, which hampers progress in related fields. This study focuses on the Poisson's ratio of a hexagonal lattice elastic network as it varies with structural deformation. By employing the Kolmogorov–Arnold Network (KAN), the transition of the network's Poisson's ratio from positive to negative as the hexagonal structural element shifts from a convex polygon to a concave polygon was accurately predicted. The KAN provides a clear mathematical framework that describes this transition, revealing the connection between the Poisson's ratio and the geometric properties of the hexagonal element, and accurately identifying the geometric parameters at which the Poisson's ratio equals zero. This work demonstrates the significant potential of the KAN network to clarify the mathematical relationships that underpin physical responses and structural behaviors.

## Graphical abstract

The present study underscores the remarkable capabilities of the Kolmogorov‐Arnold Network (KAN) in formula fitting for physical systems. KAN predicts the relationship between the Poisson's ratio of a metamaterial structure and its geometric parameters, demonstrating the transition of Poisson's ratio from positive to negative as the hexagonal component changes shape, illustrating its adaptability in analyzing complex systems.

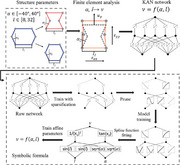

## 1 Introduction

The application of artificial intelligence in scientific research to uncover the mechanisms underlying physical phenomena represents a significant trend in future research.^[^ ^1^, ^2^, ^3^ ^]^ AI enhances scientists' ability to address complex problems, improves research efficiency, and facilitates predictions that are challenging to achieve through traditional theoretical approaches.^[^ ^4^, ^5^, ^6^ ^]^ Deep learning algorithms are extensively utilized in the scientific domain, enabling tasks such as data dimensionality reduction,^[^ ^7^, ^8^, ^9^ ^]^ dimensional expansion,^[^ ^10^ ^]^ inverse design of metamaterials,^[^ ^11^, ^12^, ^13^, ^14^ ^]^ and predictions of RNA and protein structures.^[^ ^15^, ^16^, ^17^ ^]^ Moreover, various neural networks have been employed in scientific research, including Graph Neural Networks,^[^ ^18^, ^19^ ^]^ Convolutional Neural Networks,^[^ ^20^, ^21^, ^22^ ^]^ Recurrent Neural Networks,^[^ ^23^, ^24^ ^]^ Generative Adversarial Networks,^[^ ^25^, ^26^, ^27^ ^]^ and Physics‐Informed Neural Networks.^[^ ^28^, ^29^, ^30^ ^]^

The Multi‐Layer Perceptron (MLP), grounded in the Universal Approximation Theorem, is a fundamental component of contemporary deep learning models and serves as the model for approximating nonlinear functions in machine learning.^[^ ^31^, ^32^, ^33^, ^34^ ^]^ By connecting multiple nodes, the MLP integrates nonlinear models and produces outputs, facilitating automatic learning from data for analysis and prediction. Current research on “AI for Science” often involves using neural networks to reduce dimensionality and extract features from complex data associated with physical phenomena, thereby enabling accurate predictions of their behavior based on the reduced data.^[^ ^35^ ^]^ However, key features within neural networks are obscured within the hidden layers, preventing researchers from understanding them.^[^ ^36^ ^]^ This presents two critical issues: first, the unintelligibility of these AI tools limits our ability to comprehend the mechanisms underlying physical phenomena, highlighting a lack of interpretability.^[^ ^37^, ^38^, ^39^ ^]^ Second, for the academic community to utilize trained neural networks for new predictions, relevant algorithm programs must be supplied, which restricts research findings from being independent of the computational environment, neural networks, and data. This situation hinders the dissemination of knowledge, revealing a deficiency in the communicability of the results.

In academic exploration over the past few centuries, mathematical formulas have served as the primary carriers of natural science knowledge due to their interpretability and communicability. Mathematical expressions are powerful tools that enable mechanistic analysis, facilitate predictions, and enhance the dissemination of knowledge.^[^ ^40^, ^41^, ^42^ ^]^ Consequently, transforming the feature engineering of black box models into mathematical formulas is a critical step in advancing AI for Science.^[^ ^43^ ^]^

The Kolmogorov–Arnold Network (KAN) is a deep learning algorithm based on the Kolmogorov–Arnold representation theorem,^[^ ^44^ ^]^ which states that any multivariate continuous function can be represented as a finite composite function formed by univariate continuous functions and binary addition operations.^[^ ^45^, ^46^ ^]^ KAN extends this theorem to arbitrary widths and depths, improving both accuracy and interpretability. KAN combines the strengths of MLP and spline functions, employing MLP for external feature learning and spline fitting at internal nodes. Unlike MLP that utilizes fixed activation functions, KAN replaces weight parameters at the nodes with nonlinear parametric spline functions on the edges, allowing nodes to perform only summation operations. This modification enhances both accuracy and interpretability. KAN with fewer layers achieve accuracy comparable to or exceeding that of larger MLP in function‐fitting tasks, while their symbolic regression capabilities enable more intuitive visualization.

Splines are highly precise for low‐dimensional functions and allow localized adjustments. However, they suffer from the curse of dimensionality (COD) due to their inability to leverage compositional structures. In contrast, MLPs are less affected by COD due to feature learning but are less accurate than splines in low‐dimensional cases. By integrating these approaches, KAN mitigates COD and achieves strong symbolic regression capabilities, enabling precise and interpretable mathematical formula fitting. Consequently, KAN not only predicts physics quantity like neural networks but also facilitates symbolic regression efficiently and at low computational cost, summarizing physical phenomena into an “empirical formula.”^[^ ^44^ ^]^ Mathematical formulas not only enhance our understanding of the physical mechanisms underlying phenomena but also serve as practical tools for related predictions. Thus, KAN presents new opportunities for “AI for Science”.

**Figure 1:**
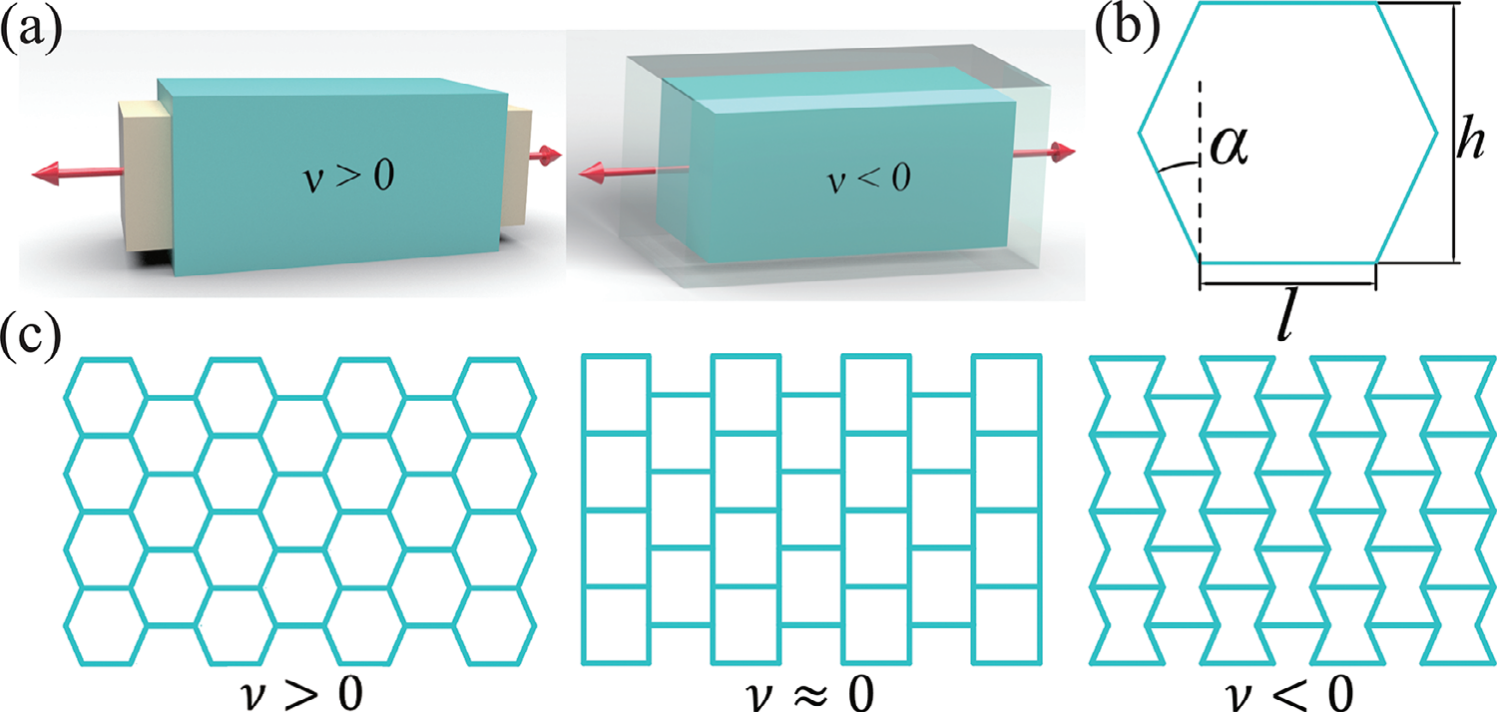
a) Schematic representation of the deformation of bulk material with both positive and negative Poisson's ratios. The initial geometry of the bulk is depicted in green, with arrows indicating the direction of the applied force. Beige represents the final state of the material after deformation, showcasing a positive Poisson's ratio in response to external loading. Conversely, transparent gray illustrates the ultimate state of the material exhibiting a negative Poisson's ratio. b) Hexagonal lattice mechanical network with a positive Poisson's ratio, where the geometric parameters include angle α, base length *l*, and overall height *h*. c) Three typical hexagonal lattice elastic network structures demonstrating positive, zero, and negative Poisson's ratios.

Despite the advantages of KAN, specific physical application cases to validate its practical utility are currently lacking. The Poisson's ratio (ν) represents the ratio of transverse negative strain to axial positive strain in materials under uniaxial tension or compression (see **Figure** **1**a).^[^ ^47^ ^]^ Previous studies indicate that modifying the geometric structure of elements in a hexagonal lattice elastic network can significantly affect the network's Poisson's ratio.^[^ ^48^, ^49^, ^50^ ^]^ As the edges of the hexagonal lattice transition from convex to concave, the Poisson's ratio shifts from positive to negative. This transition has been validated through finite element simulations and experiments.^[^ ^48^, ^51^, ^52^ ^]^ Deriving a functional expression to describe this structural effects leading to changes in Poisson's ratio is both challenging and complex, due to the bending and shearing effects in real fiber elastic network.^[^ ^48^, ^53^ ^]^

Based on this research gap, we selected the hexagonal lattice elastic network to evaluate the application potential of KAN in exploring physical mechanisms and engineering structural design. Through finite element analysis, we generated data on the relationship between the geometric parameters of the hexagonal lattice network‐angle α and base length *l* (see Figure 1b)‐and the Poisson's ratio ν. We then employed KAN to model the Poisson's ratio of the elastic network, resulting in a corresponding mathematical expression. The coefficient of determination (*R* ^2^) of this expression with the actual variation curve reached 0.986, accurately reflecting the relationship between the angle and the Poisson's ratio, thereby demonstrating excellent interpretability and accuracy. This study exemplifies the use of KAN to investigate the physical mechanisms underlying phenomena.

## 2 Results

To create the training dataset, we generated numerous elastic network structures by thoroughly scanning the geometric parameters of the hexagonal lattice elements, i.e., α and *l*. Through finite element simulations, we calculated the corresponding Poisson's ratio values for various combinations of these parameters, under the periodic boundary conditions. The range for angle α spans from −40° to 40°, the length *l* is [8, 32] mm, and the overall height *h* is 8 mm. We acknowledge the overlapping at internal concave angles when *h*/*l* ⩽ tan α, resulting in structural failure. The range for angle α spans from −40° to 40°. The length *l* falls within the range of 8–32 mm, and the overall height, *h*, is set at 8 mm. We acknowledge the presence of internal concave angle overlap when the ratio of *h* to *l* is less than or equal to the tangent of α, leading to structural failure. When the side edge rotates in a clockwise direction relative to the *y*‐axis, the angle α is considered positive, resulting in the formation of a concave polygon. Conversely, a counterclockwise rotation of the side edge results in a negative α, leading to the creation of a convex polygon. As α varies from −40° to 40°, the Poisson's ratio transitions from positive, through zero, to negative.

**Figure 2:**
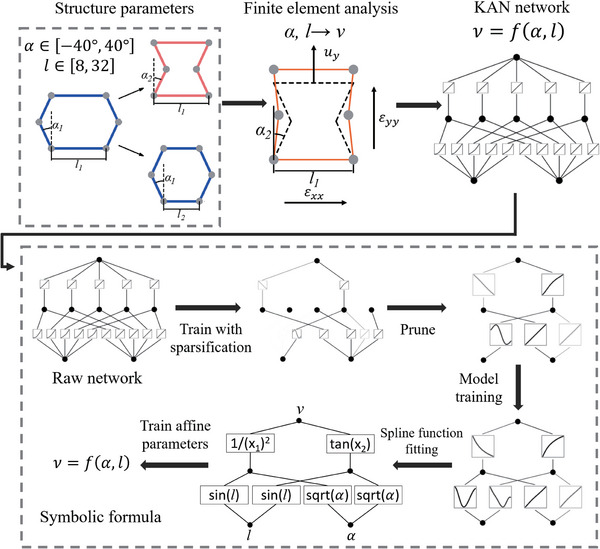
The prediction process of Poisson's ratio variation in the hexagonal lattice elastic network, considering changes in geometric parameters using finite element analysis and KAN: first, structural models are generated by varying the angle α and length *l* of the hexagonal lattice; then, a parametric scan through finite element analysis produces a dataset of Poisson's ratio ν as a function of angle α and length *l*; finally, the KAN network is trained using the constructed dataset to derive a mathematical expression.

The flowchart for training the KAN is shown in **Figure** **2**. It is crucial to adjust the dimension of the KAN to an appropriate scale. An underscaled KAN may result in the underfitting problem, while a too complicated KAN structure not only consumes computational resources, but also diminishes interpretability and accuracy, leading to overfitting. To identify the best‐performing KAN, we began with a larger structure and employed pruning adjustments based on prior knowledge of the hexagonal lattice, ultimately arriving at an optimal four‐layer KAN structure. Besides, grid size *G*, which represents the number of grid intervals used in spline function fitting, also influences accuracy and interpretability: low grid size may reduce the expressiveness of each activation function, leading to ensemble strategies that complicate interpretation, while high grid size can impede computation speed. After evaluation, grid sizes of 300 and 600 were selected.

Reducing the number of activation functions results in a concise mathematical expression for the targeted quantity. This is achieved through sparse regularization in KAN, controlled by two hyperparameters, λ and λ_*ent*_. Specifically, λ regulates the overall sparsity penalty, while λ_*ent*_ adjusts the entropy penalty, optimizing the number of active activation functions.[]

After training the KAN structure, model pruning is performed to reduce its size. Neurons with low weights are removed based on predefined thresholds, decreasing parameters and computation while preserving accuracy. The default pruning threshold is 10^−2^, with smaller values retaining more hidden nodes. Pruning also removes associated connections. Once pruned, the network is retrained to enhance performance. Upon achieving the target accuracy, spline fitting is applied to the KAN. Spline curves, defined by piecewise polynomials, are characterized by their order, *k*, typically set to 3 to balance smoothness and precision. The activation functions for the input and hidden layers are then adjusted based on the spline fitting results, generating a function combination. Fine‐tuning the coefficients of this combination further improves precision, yielding the final functional expression.

**Figure 3:**
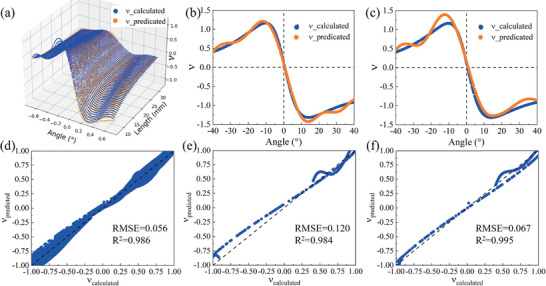
ν_*calculated*_ and ν_*predicted*_ represent the finite element simulation value and KAN prediction value respectively. a,d) Comparison of ν_*calculated*_ and ν_*predicted*_ as both the angle α and length *l* vary. b,e) The comparison between ν_*calculated*_ and Equation (2), which is derived from the KAN predicted Equation (1) under a constant bottom edge length of 8 mm. c,f) Comparison between the ν_*calculated*_ and Equation (3), which was obtained from the KAN prediction by training with the original dataset at a fixed length of *l* = 8 mm. The dashed lines in panels (d–f) represent the reference curve *y* = *x*; a closer alignment of the blue line to the dashed line indicates a greater accuracy of ν_*predicted*_ compared to ν_*calculated*_.

The trained KAN comprises three layers, with a two‐dimensional input layer corresponding to the input parameters α and *l*, and a one‐dimensional (1D) output layer. The grid size was set to 300. The prediction performance of the KAN for ν is depicted in **Figure** **3**a. The *x*‐axis and *y*‐axis represent the angle and length, respectively, while the *z*‐axis showcases the Poisson ratio values. To quantify the accuracy of the model predictions, we calculated the coefficient of determination *R* ^2^ and the root mean square error (*RMSE*) to assess the regression performance, defined a as $R^{2}=\frac{\sum {\left(\left(\hat{y_{i}}\right)-\bar{y}\right)}^{2}}{\sum {\left(y_{i}-\bar{y}\right)}^{2}}$ and $RMSE=\sqrt{\frac{\sum {\left(\left(\hat{y_{i}}\right)-y_{i}\right)}^{2}}{n}}$. The KAN predictions were compared with the finite element calculation values, as shown in Figure 3d. The predicted Poisson ratio achieved an *R* ^2^ value of 0.986 and an *RMSE* of 0.056, indicating good accuracy. Additionally, the function expression for the Poisson ratio in relation to the length *l* and angle α is provided in Equation (1).

(1) $$\begin{array}{cc} \nu = & -6.0\sin\left(\frac{0.11}{{\left(0.06x_{1}+0.25x_{3}+1\right)}^{2}}\right.\\ & \left.+0.34{\left(-0.61x_{2}+0.33x_{4}-1\right)}^{3}+1.21\right)\\ & -0.6\sin\left(-0.18{\left(0.14x_{1}+0.62x_{3}-1\right)}^{3}\right.\\ & \left.+0.08\sin(1.37x_{2}-0.74x_{4}+2.07)+4.42\right)+5.11 \end{array}$$

where *x*_1_ = sin (8.92α + 8.61), *x*_2_ = sin (2.45α − 10.08), *x*_3_ = tan (6.3*l* − 4.39), and $x_{4}=\sqrt{0.15l-1}$.

We further simplified the model to validate the accuracy and interpretability of the formula provided by KAN. As shown in Figure 3a, when the angle α varies, particularly during its transition from negative to positive, the hexagonal unit shifts from a convex to a concave polygon, resulting in the Poisson ratio changing from positive to negative, with ν = 0 at α = 0. To illustrate this phenomenon more clearly, we fixed the bottom edge length at *l* = 8 and plotted the variation of ν with respect to α in Figure 3b. The mathematical expression provided by KAN effectively elucidates the relationship between the Poisson ratio of the hexagonal lattice mechanical network and the geometric structure of the hexagonal unit. In contrast to previous vague descriptions regarding the transition of the Poisson ratio, the formula given by KAN offers a clear explanation and prediction.

By assigning a length *l* of 8 in the KAN prediction Equation (1), we derive Equation (2). The comparison between the predicted values and the computed values of this functional relationship is illustrated in Figure 3b,e. The prediction results yield an *R* ^2^ value of 0.984 and an *RMSE* of 0.120.

(2) $$\begin{array}{cc} \nu = & -6.0\sin\left(\frac{0.46}{{\left(0.12x_{1}+1\right)}^{2}}\right.\\ & \left.+0.21{\left(-0.72x_{2}-1\right)}^{3}+1.21\right)\\ & -0.6\sin\left(-2.1{\left(0.06x_{1}-1\right)}^{3}\right.\\ & \left.+0.08\sin(1.37x_{2}+1.74)+4.42\right)+5.11 \end{array}$$

where *x*_1_ = sin (8.92α + 8.61) and *x*_2_ = sin (2.45α − 10.08).

Subsequently, we trained another KAN network to derive the equation for ν and compared it with Equation (2). A 3‐layer KAN network was employed, featuring a 1‐dimensional input layer only for α, a 1D output layer, and five nodes in each hidden layer. Additionally, the grid size was set to 600. The predictive performance of the single‐variable regression for α is showcased in Figure 3c,f. In these figures, the *x*‐axis represents the angle, while the *y*‐axis represents Poisson's ratio.

(3) $$\begin{array}{cc} \nu = & -4.25{\left(1-0.07{\left(-0.97\alpha -1\right)}^{2}\right)}^{4}\\ & -0.63{\left(-\sin\left(5.34\alpha +6.89\right)-0.42\right)}^{2}\\ & +0.2\sin(5.72{\left(-0.93\alpha -1\right)}^{2}+4.46)\\ & -1.81\arctan(1.47\arctan(3.43\alpha -0.2)-0.92)\\ & -0.33\arctan(0.91\arctan(3.9\alpha -0.35)-0.03)+2.28 \end{array}$$

The KAN predicted formula for the single variable case has an *R* ^2^ value of 0.995 and an *RMSE* of 0.067. Compared to the two‐variable case, the KAN formula for the single variable demonstrates superior accuracy. The resulting function expression for the angle and Poisson's ratio is presented in Equation 3.

As shown in Figure 3, both bivariate and univariate predictions effectively capture the transition of the Poisson's ratio from positive to zero and then to negative as the hexagonal unit cell shifts from a convex to a concave polygon. While the prediction accuracy of the bivariate predictions is slightly lower than that of the univariate case, the function curves from KAN predictions across different network structures and dataset sizes exhibit consistent trends. The results suggest that the KAN‐derived mathematical expression aligns more closely with reality under fewer parameters. However, a more complex case with numerous parameters is required to investigate the correlation between the KAN's empirical formula and physical phenomena, as well as the impact of parameter quantity. This analysis lies beyond the scope of the present study. The *R* ^2^ values for the predictions are 0.995 and 0.986, indicating excellent accuracy. Notably, the position of the zero Poisson's ratio, which marks the transition between positive and negative values, is predicted with high precision. Additionally, the predictive formula derived from KAN comprises functional forms that include trigonometric and power functions, consistent with the fundamental physical principle that the Poisson's ratio of the mechanical network is determined by its geometric structure. KAN bridges the gap between traditional neural networks and symbolic computation. Its ability to derive explicit formulas supports knowledge dissemination, enhancing communicability and independence from specific computational environments. This property is particularly valuable for scientific applications requiring both precision and understanding. KAN' spline‐based architecture allows it to seamlessly handle tasks ranging from high‐dimensional data exploration to low‐dimensional functional modeling. Our results show that Kan exhibits high accuracy and interpretability on small‐scale AI for scientific tasks.

## 3 Conclusion

Based on finite element simulations and KAN network predictions, we successfully predicted the Poisson's ratio for the hexagonal lattice mechanical network and derived its functional relationship. KAN accurately established that as the hexagonal unit structure transitions from a convex to a concave polygon, the Poisson's ratio shifts from positive to negative values. The function expression provided by KAN comprises trigonometric and power functions, reflecting the influence of geometric parameters on the Poisson's ratio.

Furthermore, when analyzing physical mechanisms with small datasets, the KAN network requires fewer computational resources than traditional machine learning algorithms. In our study, calculations were conducted using only a CPU, demonstrating that this method can effectively predict physical properties under typical computational conditions. Additionally, the generated mathematical formulas possess explanatory power, elucidating the underlying physical mechanisms and facilitating knowledge dissemination within the academic community. KAN uniquely transforms complex physical phenomena into symbolic mathematical expressions, making it well‐suited for small‐scale AI for science tasks. For instance, KAN effectively models the relationships between thermal conductivity, elastic modulus, and other properties with thermodynamic quantities. Future work will further validate the KAN network in this context. In summary, we emphasized the significance of KAN's accuracy and interpretability in engineering design and the study of physical mechanisms, using the case of the Poisson's ratio in hexagonal lattice networks as a focal point.

## 4 Experimental Section

The Kolmogorov–Arnold network is a machine learning algorithm derived from the Kolmogorov–Arnold representation theorem.^[^ ^45^, ^46^ ^]^ In real analysis and approximation theory, this theorem asserts that a multivariate continuous function can be expressed as a finite composition of single‐variable continuous functions and binary addition operations. Specifically, for a smooth function $f:{\left[0,1\right]}^{n}\to R$

(4) $$f\left(x\right)=f\left(x_{1},\;\text{…},x_{n}\right)=\sum_{q=0}^{2n}\Phi_{q}\;\left(\sum_{p=1}^{n}\phi_{q,p}\;\left(x_{p}\right)\right)$$

where $\phi_{q,p}:\left[0,1\right]\to R$ and $\Phi_{q}:R\to R$.

This indicates that the sum is the only genuinely multivariate function, as all other functions can be expressed through univariate functions and summing. However, the expressive power of a neural network based on the Kolmogorov‐Arnold representation theorem, limited to a depth of two layers and a width of (2n+1), may lead to less smooth prediction curves.^[^ ^54^, ^55^ ^]^ The KAN generalizes this representation to accommodate arbitrary width and depth, thereby extending beyond the constraints of the original two‐layer, (2n+1) width fitting structure.

Unlike MLP, which applies manually adjusted activation functions at nodes, KAN assigns learnable activation functions to the edges.As a result, KAN does not possess a linear weight matrix; each weight parameter is substituted with a learnable 1D function parameterized as a spline. KAN nodes simply sum the inputs without applying non‐linearity. Essentially, KAN integrates spline functions with MLP characteristics, leveraging the advantages of both approaches. This enables KAN to learn features that benefit from high‐dimensional expansion while also optimizing these features with high precision in lower dimensions. When employing KAN for data fitting, the dataset is first input into the KAN, where sparse regularization trains the model to obtain weights for each activation function. Subsequently, the trained KAN structure is pruned by removing “unimportant” activation functions, and refining the model to an appropriate size. After additional training to achieve the target accuracy, spline function fitting is applied to each activation function of the KAN network to derive symbolic expressions.

Nevertheless, KAN has potential limitations. As the dimensionality of the input parameter space increases, the computational efficiency of KAN may decline due to the rising complexity of spline‐based activation functions. This issue becomes pronounced in large datasets with complex nonlinear interactions among numerous variables. Although pruning and sparse regularization can alleviate overfitting, these techniques may also restrict the model's generalization capacity in high‐dimensional settings.

In this study, a Gen Intel(R) Core (TM) i5‐13400 CPU was used for finite element simulations conducted with COMSOL 6.2. Geometric models are generated by varying the global parameters, angle α and length *l*. Periodic boundary conditions along the symmetric edges of the hexagonal lattice simulate its deformation behavior, eliminating edge effects and capturing the intrinsic mechanical properties. A uniaxial tensile displacement of 2 mm is applied along the *y*‐axis to remain within the material's elastic limit. The mesh density was set to an ultrafine size, controlled by the physical field network, ensuring a balance between precision and computational efficiency. The mesh comprised 536 domain elements and 268 boundary elements. The Poisson's ratio is calculated from the ratio of periodically averaged strain probes in the unit cell. Linear elastic material properties were selected to match the mechanical characteristics of commonly used 3D printing resin materials. The material parameters for the structure were set as follows: Young's modulus *E* = 1.2 × 10^7^ *Pa*, Poisson's ratio ν = 0.2, and density ρ = 1300 *kgm* ^−3^. For structures with varying angles and lengths, a training set of 39 100 samples was generated through finite element simulations and divided into training, testing, and validation sets in an ≈3:1:1 ratio.

In the KAN network with dual parameter variations in angle and length, the KAN layer is configured with a width of [2, 2, 2, 1], a grid size of 300, k = 3, and a random seed of 114514. The optimization algorithm employed in the training model is LBFGS, with λ set to 0.01 and λ_*ent*_ set to 0.00. Appropriate symbolic functions for the activation functions are automatically selected from a library that includes *x*, *x* ^2^, *x* ^3^, *x* ^4^, $\frac{1}{x}$, $\frac{1}{x^{2}}$, $\frac{1}{x^{3}}$, $\frac{1}{x^{4}}$, $\sqrt{x}$, $\frac{1}{\sqrt{x}}$, *e* ^*x*^, sin *x*, tan *x*, arcsinx, arctanx. During coefficient training for the spline functions, the optimization algorithm remains LBFGS, with a truncation step of 50.

In the KAN network with a single parameter variation in angle, the KAN layer is set with a width of [1, 5, 1], a grid size of 600, k = 3, and a random seed of 114514. The optimization algorithm used in this training model was also LBFGS, with λ set to 0.01 and λ_*ent*_ set to 0.00. Similarly, the appropriate symbolic functions for the activation functions are selected from a library that includes *x*, *x* ^2^, *x* ^3^, *x* ^4^, $\frac{1}{x}$, $\frac{1}{x^{2}}$, $\frac{1}{x^{3}}$, $\frac{1}{x^{4}}$, $\sqrt{x}$, $\frac{1}{\sqrt{x}}$, *e* ^*x*^, sin *x*, tan *x*, arcsinx, arctanx.

## Conflict of Interest

The authors declare no conflict of interest.

## Data Availability

The data that support the findings of this study are available from the corresponding author upon reasonable request.
